# Tracking the current in the Alzheimer's brain - Systematic differences between patients and healthy controls in the electric field induced by tDCS

**DOI:** 10.1016/j.ynirp.2023.100172

**Published:** 2023-04-26

**Authors:** Ingrid Daae Rasmussen, Matthias Mittner, Nya Mehnwolo Boayue, Gábor Csifcsák, Per M. Aslaksen

**Affiliations:** a Department of Psychology, Research Group for Cognitive Neuroscience, Faculty of Health Sciences, UiT the Artic University of Norway, Tromsø, Norway; b Department of Geropsychiatry, University Hospital of North Norway, Norway; c Department of Child and Adolescent Psychiatry, University Hospital of North Norway, Tromsø, Norway

**Keywords:** tDCS, Transcranial direct current stimulation, Alzheimer's disease, Computational modeling, tDCS-induced electric field, Noninvasive brain stimulation

## Abstract

**Background:**

Several studies on patients with Alzheimer's disease (AD) have used transcranial direct current stimulation (tDCS) to enhance neural excitability in the left dorsolateral prefrontal cortex (lDLPFC). Interindividual differences in brain anatomy in AD patients pose a challenge to efficiently target the lDLPFC using scalp-based coordinates, calling for new and more precise tDCS protocols.

**Objective:**

The purpose of this study was to explore how AD-related neuropathology affects the tDCS-induced electric field (EF) across different DLPFC montages using computational modeling.

**Method:**

Forty-eight realistic head models were created from structural magnetic resonance scans of AD patients and healthy controls collected from a publicly available database. We compared the tDCS-induced EF in different montages applied in the literature, in addition to a high definition (HD)-tDCS montage centered at electrode F3.

**Results:**

There was an overall global reduction in EF strength in the patient group, probably due to structural alterations that were also identified in the patient group. A widespread distribution of the EF was found across the frontal lobe for bipolar montages, while HD-tDCS yielded more focal stimulation, mainly restricted to the lDLPFC. Minor differences in the EF distribution were found when comparing the HD-tDCS montages.

**Conclusion:**

Neurodegenerative alterations present in patients with AD affect the magnitude, distribution and variability of the EF. HD-tDCS montages provide more focal stimulation of the target area, compared to bipolar montages with to pronounced group differences between AD patients and healthy matched controls. This finding poses substantial limitations to the comparison of cognitive effects of tDCS both between patients and controls and within patients at different stages of disease progression.

In Alzheimer's disease (AD), neural activity is severely affected by neurodegenerative processes (Frisoni et al., 2010). By applying electric current to brain regions associated with memory performance, several studies have aimed to facilitate neural connections and enhance memory function for these patients using transcranial direct current stimulation (tDCS) (Cai et al., 2019; Chang et al., 2018).

The first tDCS studies on AD patients reported optimistic results, showing that tDCS improved patients’ performance on recognition memory tasks (Boggio et al., 2009, 2012; Ferrucci et al., 2008). However, the following decade yielded rather mixed results (Bystad et al., 2016; Cotelli et al., 2014) challenging the therapeutic potential of tDCS in AD. Cappon et al. (2016) highlighted the diversity of the methodological approaches used in the field of tDCS in cognitive rehabilitation: Studies targeted different cognitive functions, with variable current intensities, electrode dimensions and stimulation durations. Clearly, the application of more standardized protocols is necessary to provide sufficient evidence for the effectiveness of this intervention, before clinical guidelines can be made (Lefaucheur et al., 2017). For the purpose of optimizing stimulation protocols, we first need to identify the main sources of variability. Here, we propose that computational modeling can help transition from incidental parameters such as electrode size and location and focus instead on the active component of the method, the intensity of the electric field (EF) in the target area.

Computational modeling enables the prediction of the magnitude and spatial distribution of tDCS-induced EF in the brain, providing crucial insights into the neural mechanisms and associated behavioral outcomes of this brain stimulation technique (Bikson et al., 2012; Mahdavi and Towhidkhah, 2018; Opitz et al., 2015). In addition to protocol-related factors such as electrode size, positioning and current intensity, interindividual differences in head and brain anatomy also influence the flow of tDCS-induced current (Antonenko et al., 2020; Datta et al., 2012; Laakso et al., 2016; Opitz et al., 2015).

In the AD population, there are interindividual differences in the degree of brain atrophy at different stages of the disease (Hill et al., 2011). As AD progresses, the loss of neurons and synaptic injury results in both larger ventricular areas and a reduction in gray matter (Frisoni et al., 2010). Increased cerebrospinal fluid (CSF) volume affects the current pathways (Bikson et al., 2012; Datta et al., 2012), which in turn can substantially influence tDCS outcomes in patients. Therefore, placing electrodes based on fixed coordinates on the skull does not guarantee that the target brain area receives sufficiently strong currents (Opitz et al., 2015), which indicates a need for more precise montage optimization. In addition, electrode placement in AD patients has been informed by studies on the cognitive effects of tDCS in healthy individuals. However, due to significant differences in anatomy between the AD brain and normal aging, regarding gray matter atrophy, white matter damage and hippocampal volume loss (Toepper, 2017; Vemuri and Jack, 2010; Fjell and Walhovd, 2010), both the distribution of the EF and the behavioral outcomes of tDCS can differ relative to the healthy brain. By quantifying the magnitude and spatial distribution of EF in the brains of AD patients, we can adjust the stimulation protocol to optimize cortical targeting. More precise stimulation is likely to increase the chances of treatment success (Mahdavi et al., 2014).

Bipolar montages are the most common tDCS protocols, consisting of one anode and one cathode electrode. In AD montages, the anode is often placed either above the left dorsolateral prefrontal cortex (lDLPFC) (Boggio et al., 2012; Im et al., 2019; Khedr et al., 2014; Penolazzi et al., 2015) or on the medial temporal lobe (Boggio et al., 2009; Bystad et al., 2016; Ferrucci et al., 2008), whereas the cathode is typically positioned above the right hemisphere. Bipolar montages result in approximately 50% of the induced current reaching the cortex (Nitsche et al., 2015), while the rest is shunted away. These montages are nonfocal, causing widespread currents outside the target area (Datta et al., 2012; Opitz et al., 2015), a phenomenon that can severely confound the interpretation of the cognitive or clinical effects of these protocols (Csifcsák et al., 2018).

More recently, high definition-tDCS (HD-tDCS) has been introduced (Datta et al., 2012). This stimulation protocol uses a “4 × 1 layout” consisting of an anode placed above the target area surrounded by four return electrodes (cathodes). The ring-shaped electrodes are smaller than the conventional bipolar ones, usually 1.2 cm in diameter versus the rectangular 5 × 7 cm electrodes. The 4 × 1 ring montage increases spatial focality (Alam et al., 2016; DaSilva et al., 2015). The 4 × 1 montage was used in our recently published study, where patients receiving active HD-tDCS improved significantly on delayed memory tasks compared to patients receiving sham tDCS (Rasmussen et al., 2021). Importantly, electrode positioning in this study was informed by computational modeling of the EF. However, to draw conclusions on the efficacy of HD-tDCS montages in the AD population and on the utility of individual montage optimization, a more systematic comparison between bipolar and HD-tDCS montages is needed.

In the present study, the strength and spatial distribution of tDCS-induced EF in 48 MRI-derived realistic head models were analyzed. The aim was to compare EF distributions from six different electrode montages targeting the lDLPFC and to explore the effect of anatomical variations on the EF, with special emphasis on AD-associated brain atrophy. Four bipolar montages and one HD-tDCS montage targeting the lDLPFC, which have been previously applied in the literature, were analyzed (Table 2) in addition to an standard F3 HD-tDCS montage.

We hypothesized that atrophy in the AD brains would result in more variability in the EF for all montages. Furthermore, we hypothesized that HD-tDCS would result in an EF that is more constrained to the target region than the standard bipolar positioning approach, and that the individual optimization would increase the strength of EF in the lDLPFC. Due to AD-related pathology, we also anticipated that optimized electrode placement would be more beneficial in the AD group in terms of restricting the EF to the target area. To our knowledge, there are no previous modeling studies of this nature that compared patients with diagnosed AD and healthy matched controls.

## 1 Methods and materials

### 1.1 Participants and MRI acquisition

High-resolution head models were created from T1-and T2-weighted anatomical images collected from the OASIS-3 study in the XNAT database (http://www.oasis-brains.org). The OASIS-3 is a longitudinal neuroimaging, clinical, cognitive, and biomarker dataset for normal aging and AD. Structural MRI scans of 24 AD patients (13 women; mean ± SD age: 72.05 ± 5.49) and an equal number of healthy, matched controls (14 women; mean ± SD age: 70.36 ± 2.20) were used (Table 1).

**Table 1:** Clinical and demographic data.

Variable	Alzheimer (N = 24)	Healthy (N = 24)	*t value*	*p value*
Sex male/female (N)	11/13	10/14	–	–
Age (M ± SD)	72.05 ± 5.49	70.36 ± 2.20	1.40	.169
Education in years (M ± SD)	14.96 ± 2.79	16.42 ± 2.67	−1.851	.071
MMSE-NR (M ± SD)	17.04 ± 4.90	29.71 ± 0.46	−12.60	<0.01*

Note. Independent T-test. M: mean, SD: standard deviation, MMSE-NR: Mini Mental Status Evaluation Revised. ∗Indicates p < .05.

**Table 2:** Previous DLPFC-tDCS studies on AD patients using bipolar montages.

Study	Design	Electrode position A	Electrode position C	Electrode area cm^2^
Liu et al., (2020)	Cross- over	l&r DLPFC (F3&F4)	Inion (lz)	35
Im et al., 2019	RCT	l DLPFC (F3)	rDLPFC (F4)	36
Khedr et al. (2014)	RCT	l&r DLPFC	contralateral SOA	A: 24, C: 100
Boggio et al. (2009)	Cross- over	l DLPFC (F3)	r SOA	35

Note. 2 mA current intensity for all studies. RCT: randomized controlled trial, r: right, l: left.

DLPFC: dorsolateral prefrontal cortex, SOA: supraorbital area, A. anodal, C: cathodal.

### 1.2 tDCS simulation

The procedure for creating the head models was semiautomatic (with manual quality-control steps) using a pipeline developed in Nipype (Gorgolewski et al., 2011). Head models were created with the “mri2mesh” routine in SimNIBS, version of 2.1 (www.simnibs.org/; Thielscher et al., 2015), a software package developed for calculating the EF induced by noninvasive brain stimulation. The “mri2mesh” routine relies on FreeSurfer (Fischl, 2012) for automatic segmentation of gray and white matter and accurate cortical surface reconstruction and FSL (Smith et al., 2004) for automatic tissue segmentation of skin, skull and CSF. Segmentation quality can be checked here: https://osf.io/9wgrq/. Calculations of the tDCS-induced EF were run using the finite element method (FEM). The FEM model gives information about the EF (both intensity and distribution) based on the tDCS dose (mA), conductance of the tissues (e.g., skin, skull, CSF, white- and gray matter), head anatomy and electrode parameters (number of electrodes, their location, shape, size, thickness, and the conductive medium: gel or saline-soaked sponge sockets). The conductivity of the head tissues was based on the default settings in SimNIBS (Supplementary Table 1).

**Fig. 1:**
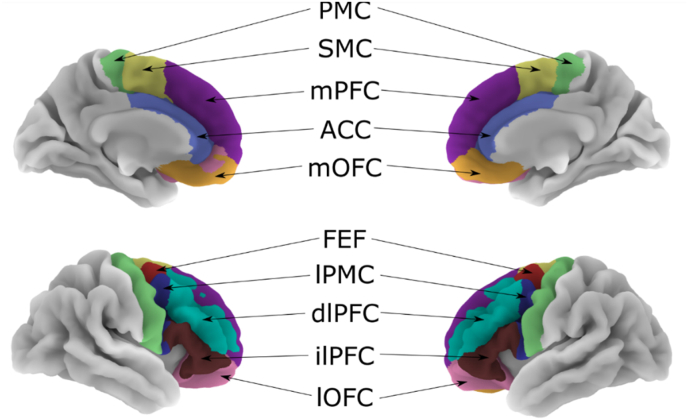
The Ranta atlas dividing the frontal lobe into ten regions per hemisphere. Note. PMC: primary motor cortex, SMC: supplementary motor complex, mPFC: medial prefrontal cortex, ACC: anterior cingulate cortex, mOFC: medial orbitofrontal cortex, FEF: frontal eye field, lPMC: lateral premotor cortex, dlPFC: dorsolateral prefrontal cortex, ilPFC: inferior lateral prefrontal cortex and lOFC: lateral orbitofrontal cortex.

Four bipolar tDCS montages and two HD-tDCS montages were simulated for each head model. The bipolar montages were sized and positioned as described in the original papers (Table 2), with an electrode thickness of 1 mm, circular connectors (diameter: 0.5 cm) at the middle of the electrode pads, and a sponge pocket of 2.5 mm. The current intensity was set to 2 mA for all montages. Both HD-tDCS montages were based on the extended 10/20 EEG system (Klem et al., 1999) with one anode electrode (2 mA) surrounded by four cathode electrodes (0.5 mA each), with electrodes of 1.2 cm diameter, thickness of 1 mm and a 2.5 mm gel thickness. In our “uniform” HD-tDCS montage, the anode was positioned at location F3, and the surrounding electrodes were placed at F7, C3, Fz and Fp1 in all head models. In the optimized HD-tDCS montage, the selection of the location of the anode was based on individual optimization of the magnitude of the EF in the target area (lDLPFC), derived from computational modeling. This optimization approach was recently used in our randomized pilot study involving patients with AD (Rasmussen et al., 2021). More specifically, eight different 4 × 1 montages over the DLPFC was simulated (Supplementary Figure 1 for all anode and cathode locations), where the optimal montage was chosen based on two rules. First, the highest value of the anodal current (positive values for the normal component of the EF) had to be in the lDLPFC compared to the other regions in the frontal cortex. From the montages that fulfilled this condition, the montage with the highest difference between the anodal and cathodal EF in the left DLPFC was chosen. This second rule was designed to prevent strong cathodal currents in the target area, which are associated with neural inhibition (Nitsche et al., 2003). The lDLPFC was localized using the Ranta atlas (Ranta et al., 2009, 2014), which is a parcellation of the frontal lobe into ten distinct regions in each hemisphere (see Fig. 1).

### 1.3 Data extraction

From the three-dimensional vector field quantifying the distribution of the EF (three-dimensional direction vectors for each of the finite-element nodes in three-dimensional space), we calculated four indices that were averaged within the brain regions:

1) The “normfield” measures the absolute strength of the EF at each node. This gives information about the EF intensity at that exact location, without taking the current direction (polarity) into account.
2) The “normal component” reflects currents either perpendicularly entering or leaving the cortex (positive and negative values, respectively). The current entering the cortex is commonly associated with increased neural excitability (“anodal effect”, positive values), whereas current leaving the gray matter toward the CSF is inhibitory in nature (“cathodal effect”, negative values). For both the normfield and the normal component, region- and hemisphere-specific mean and SD values were obtained.
3) A “target focality index” for both anodal and cathodal currents, defined as the proportion of nodes in the lDLPFC with peak 1% EF intensities (“hotspots”) relative to the number of hotspots in the whole cortex (Csifcsák et al., 2018).
4) The coefficient of variation in the patient and control groups to determine whether anatomical differences within groups affected the variability of the EF in the frontal lobe. The coefficient of variation was calculated as the standard deviation of the normal component divided by the mean of the normfield in each region and multiplied by 100 (Laakso et al., 2016).

We used raw EF values without any normalization.

### 1.4 Brain structure segmentation

The volume, area and thickness values of the MRI scans were provided by FreeSurfer version 6.0 software with the recon-all processing pipeline, including motion correction, normalization to Talairach space, intensity bias correction, skull stripping, surface registration and segmentation. FreeSurfer segmentation outputs were visually inspected in FreeView for severe errors (e.g., skull strip errors, segmentation errors and pial surface misplacement). No manual correction was performed. Values of the cortical thickness, volume and area in 10 frontal regions of each hemisphere were extracted from the Ranta atlas and compared using separate univariate ANOVA for each region (Fig. 1; Ranta, 2009, 2014). Volume measures were controlled for intracranial volume.

### 1.5 Analysis

To evaluate the montage- and diagnosis-specific effects (AD patients vs. healthy control subjects) on the EF magnitude and spatial distribution, we conducted sequences of hierarchical Bayesian regression models using the brms package (Bürkner, 2017). All reported analyses employ hierarchical linear models (also known as mixed-effect models) where subject-level (random-effects) and group-level (fixed effects) are combined when estimating the best-fitting model. We use Bayesian methods for estimating these models because they allow a flexible model-building process and implement advanced methods for determining effect-size estimates (using posterior means and highest-density intervals) as well as for model comparison. In all of these models, we use the EF as dependent variable (either the normal component of the EF or its non-directional intensity) and use predictor variables coding for the brain region and hemisphere (in order to account for the obvious variability in which brain regions are stimulated) as well as the montage to quantify differences between montages. Interactions between all these factors are also included in order to analyze in which region-specific montages differ from one another. Finally, and crucially, we include a factor coding for which group the participant belongs to (i.e., whether it is an AD patient or a healthy control subject). To account for inter-individual global differences in the EF (as might be caused by within-group variations of factors such as skull-thickness, for example), we added random intercepts per subject.” In total, 16 models were evaluated per analysis: a null model with no predictors, four models with a single predictor, six models for all pairs of predictors and their interactions, four models for all triplets of predictor combinations and a full model with all predictors and interactions. From this ensemble of models, we selected the best-fitting model using the leave-one-out cross-validation criterion (LOOIC; Vehtari, Gelman & Gabry, 2015). With this approach, lower LOOIC values are indicative of a better fit. All models were estimated using Hamiltonian Monte-Carlo methods (HMCs) implemented in Stan (Stan Development Team, 2016). We used four chains of 2000 samples each, where the first 1000 samples were treated as the warm-up period and discarded from the final analysis. All traces had R^^^-values below 1.05 and were visually inspected for convergence (Gelman et al., 2013). R^^^-values larger than 1.05 indicate insufficient exploration of the posterior density and would therefore prevent the interpretation of the results of the statistical model. We used the default noninformative priors implemented in brms. For all models, we report the raw regression coefficient (*b*) along with the 95% highest density interval (95% HDI), in which the true value falls with 95% probability given the validity of the model.

## 2 Results

### 2.1 Total EF-strength reduced in AD patients

The comparisons of the MRI scans showed that AD patients had a significantly thinner cortex in almost all brain regions and reduced volume compared to the healthy matched controls. In the lDLPFC the AD had a significantly thinner cortex (M = 2.26 mm, SD = 0.12) compared to the control group (M = 2.41 mm, SD(0.10), F(19.68), p < .001. For all values see Supplementary Table 2. Results of the hierarchical Bayesian regression models, testing whether this atrophy affected the total EF strength (see method section “Analysis”), showed that the model where the group effect was limited to a main effect (i.e., the effect was fixed across regions, hemispheres and montages) was preferred by the model selection (LOOIC =−30110.3, SE =156.1, $R^{2}=0.94$). The results showed that AD patients had generally reduced electric field strengths across brain regions and montages, b=−0.011, 95% HDI [−0.0024,−0.021]. For the full model-selection table, see Supplementary Table 3.

### 2.2 Greater EF variability in AD patients

We expected that EF distribution would show greater variability in AD patients given their greater anatomical variability. We therefore conducted an equivalent analysis of the coefficient of variation as in the previous section, where we included a main-effect-only model for the patient group in addition to the other 16 models, including the different predictor combinations. In this analysis, the winning model included all predictors, including patient group *and* all interactions between these factors (LOOIC =34410.3, SE =146.9, $R^{2}=0.77$). The second-best model was the one where patient group was included as a main effect only (LOOIC =34416.9, Standard Error (SE) =149.3; $R^{2}=0.76$) (Supplementary Figure 2 and Supplementary Table 4). In all areas and montages, the coefficient of variation was always increased in the AD group relative to the healthy controls (average increase: *b* = 1.56, 95% HDI [0.67, 2.43]). Therefore, we conclude that the variability of the EF was significantly affected by patient group and that the effect was different across montages, regions and hemispheres.

### 2.3 Variations in EF between bipolar- and HD-tDCS montages

**Fig. 2:**
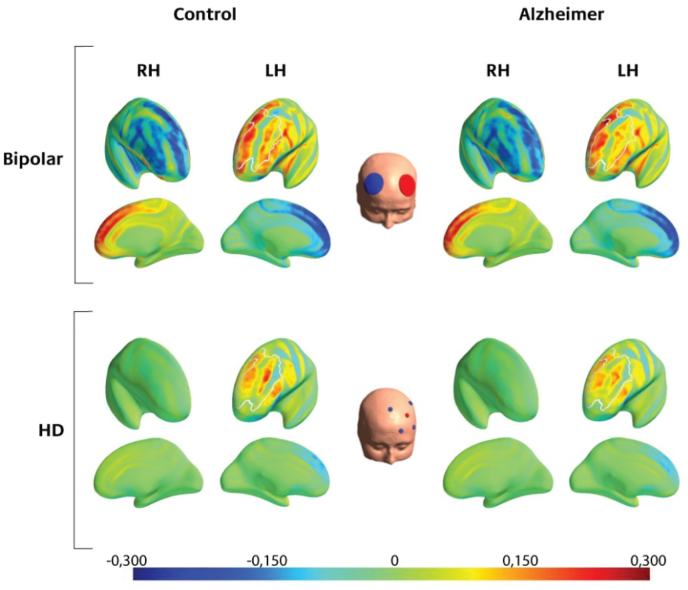
Comparison of the normal component in a bipolar and an HD-tDCS montage. Note. EF distribution for the bipolar (Im et al., 2019) and uniform HD-tDCS montage. The unit of the EF normal component is in V/m. Dark red indicates a strong inward flowing current, while dark blue represents a strong outward flowing current. For both montages, the stimulation intensity was set at 2 mA. The current in the HD-tDCS montage is more focalized, not affecting the right hemisphere. However, the anodal current in the bipolar montage is stronger.

**Fig. 3:**
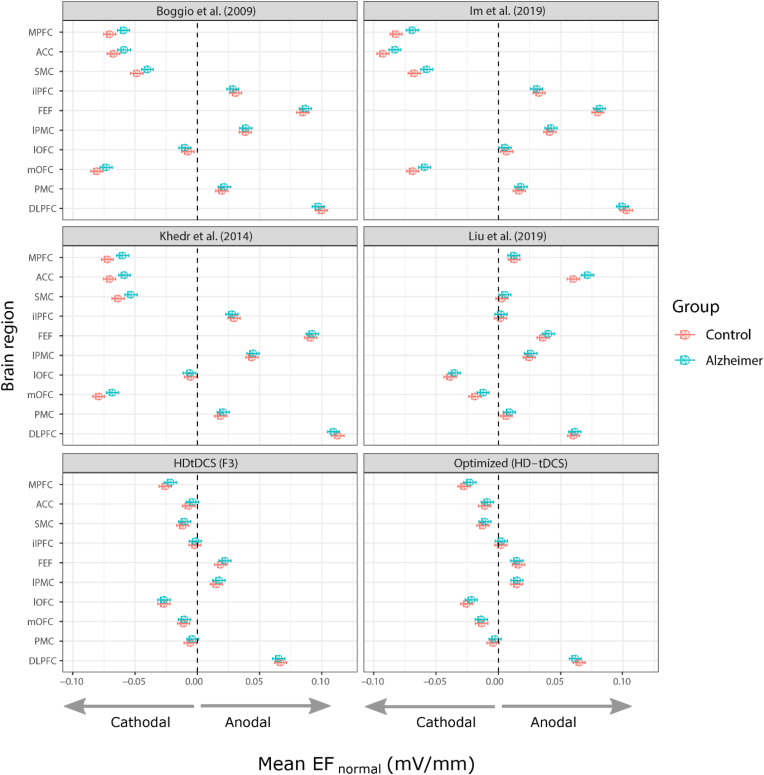
Marginal means for the normal component of the electric field in the left frontal cortex for all tDCS montages.

**Fig. 4:**
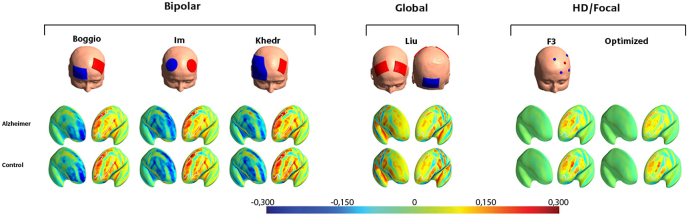
Mean of the normal component across all different montages. Note. Colorbar unit V/m. See “Table 2” for specific placements of electrodes in each montage.

To investigate the distribution of the anodal and cathodal EF, we estimated a sequence of regression models, treating the mean normal component in each brain region as the dependent variable. The best model (LOOIC =−34257.1, SE =193.3, $R^{2}=0.94$) was the full model that included all four predictors: patient group, montage, brain region and hemisphere, as well as their interactions (Supplementary Table 5). Consequently, all of these variables were predictive of the average electric field inducing anodal (positive) or cathodal (negative) currents. Fig. 2 illustrates the difference between the bipolar and HD-tDCS montages, showing both the lateral and medial aspects of the brain. Fig. 3 shows the estimated anodal and cathodal effects induced in each frontal brain region in the left hemisphere separately for the two groups. Fig. 4 illustrates the spatial distribution of the anodal and cathodal effects in both hemispheres for each montage (group means and standard deviations), separately for the patient group and the healthy matched controls.

The profiles for the three bipolar montages with the anode electrode over the left DLPFC and the cathode electrode over the right DLPFC (Boggio et al., 2009; Im et al., 2019; Khedr et al., 2014) are quite similar, and all show strong cathodal stimulation of medial frontal areas (MPFC/ACC/SMC/mOFC) as well as non-prefrontal areas (Fig. 2, Fig. 3). In contrast, the nonfocal montage used by Liu et al. (2020), with one anode electrode over each DLPFC and the cathode electrode placed over the inion, shows strong anodal stimulation of the MPFC and ACC and less stimulation in non-prefrontal areas. Finally, the optimized and F3-based HD-tDCS montages showed comparably strong EFs in the target area (left DLPFC) but largely reduced EF magnitudes in the remaining frontal structures. Group differences are most pronounced in the three bipolar montages. There does not appear to be a clear difference between healthy and AD patients when using the focalized HD-tDCS montages.

#### 2.3.1 Limited effect of optimizing the HD-tDCS montages

Following up on these results, we conducted an analysis restricted to the HD-tDCS montages. The winning model (LOOIC =−13349.7, SE =140.1, $R^{2}=0.87$) included all factors except the patient group, indicating that diverging anatomical features between the two groups did not significantly alter the induced E-field (normal component) in the HD-tDCS montages (Supplementary Table 6). However, since “montage” was included in the winning model, the optimized and F3 versions of the HD-tDCS ring-montages induced different EF distributions. Surprisingly, the average anodal EF in the target region, the left DLPFC, was slightly reduced in the optimized montage relative to the F3 montage (b=−0.0028; [−0.0052,0.0004]), even though the 95% HDI includes zero and the effect is therefore not robust.

### 2.4 Selection of electrode position in the optimized montage

**Fig. 5:**
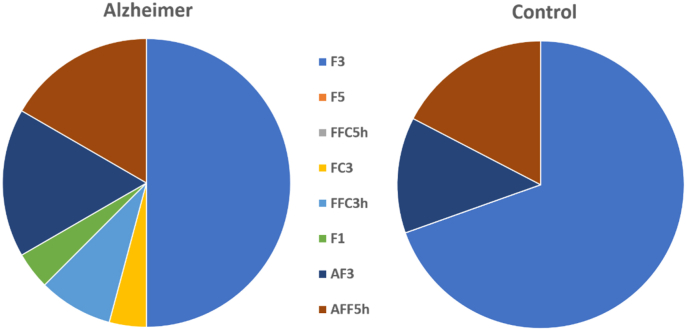
Electrode montage selection for the optimized HD-tDCS protocol. Note. Coordinates based on the 10–20 EEG system.

For the optimized montage, three different montages were chosen in the control group, while six different montages were chosen in the Alzheimer group (Fig. 5).

### 2.5 Focality of lDLPFC stimulation

**Fig. 6:**
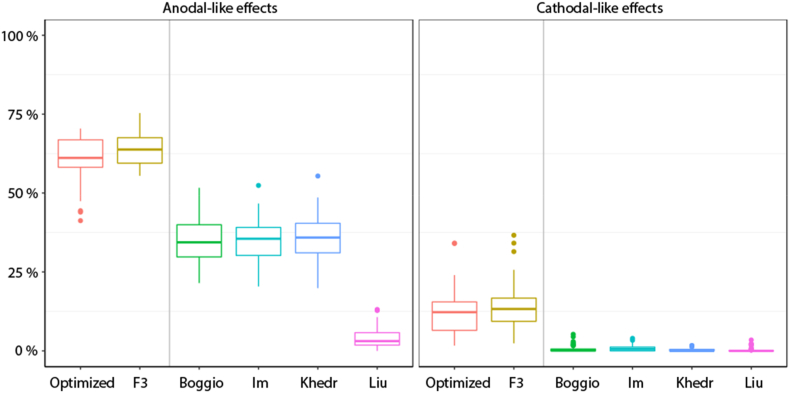
Focality index of anodal and cathodal current effects in the left DLPFC. Note. Percentage of the top 1% highest normal component EFs located in the lDLPFC relative to the whole cortex.

Focality in the lDLPFC was calculated based on the percentage of nodes with the top 1% highest normal component EFs located in the lDLPFC relative to the whole cortex. The HD-tDCS montages had the majority of high activity nodes in the target region. The three bipolar montages had approximately one-third of the high nodes in the lDLPFC, while the Liu montage had very few high-activity nodes in the target region (Fig. 6).

## 3 Discussion

The primary goal of the present study was to compare the tDCS-induced EF across different montages targeting the left DLPFC in AD patients and healthy matched controls using computational modeling. Anatomical comparison of the two groups showed a statistically significant thinner cortex and reduced cortical volume in the AD group. Computational modeling revealed a weaker EF strength in AD patients, in addition to greater variability across the frontal lobe in both hemispheres. The analysis showed widespread EF in the bipolar montages compared to the more focal stimulation in the HD-tDCS montages. In addition, the optimized and uniform F3 montage showed only minor differences in the EF distribution.

Our results show that the simulated tDCS-induced EF was weaker across all montages and brain regions for the AD group than for the control group, especially in brain regions not directly underneath the electrodes. These results are in line with previous modeling studies that have indicated that decreased gray matter and higher levels of CSF may reduce the current density (Laakso et al., 2016; Opitz et al., 2015). In a comparison study of three brain models (Mahdavi and Towhidkhah, 2018), an increase in CSF and gray matter atrophy was related to a reduced magnitude of current density. A study by Antonenko et al. (2020) also showed that older adults had higher interindividual anatomy, affecting the current density. Our study, with a total of 48 head models, is the first to show that the aging brain affected by AD neurodegeneration receives even less current density than the normal aging brain. Based on these results, generalization from tDCS studies on healthy adults to AD patients should only be done with great caution.

To successfully reach the brain region of interest, Habich et al. (2020) promote two conditions that need to be fulfilled. Primarily, the dose that reaches the target area in the cortex must be sufficient to modulate the cortical activity, and second, the current must reach the correct target. Since the current dose that reaches the AD brain is reduced, it is plausible that patients might benefit less from tDCS stimulation than healthy controls if the same intensity is administered. A possible solution to match the effective dose of the stimulation is to individualize the tDCS protocol based on the results from computational modeling, whereby the stimulation intensity is adjusted so that all patients receive the same EF values in the target area. Increasing current intensity from 2 mA to 3 mA would increase current density in the AD brain and is shown to be tolerable and without adverse side effects when using HD-tDCS (Reckow et al., 2018). However, as stated by both Mahdavi and Towhidkhah (2018) and Thomas et al. (2018), brain atrophy with increased CSF may lead to both “shunting” of current and congestion of CSF attracting current to brain regions outside of the target of stimulation. Another possible approach for optimizing tDCS is to regulate the duration of stimulation. Further studies are needed before concluding how these parameters influence treatment success (Lefaucheur et al., 2017).

The patient group showed higher variability in the EF distribution across the brain in all montages, especially in the bipolar montages. If the tDCS intensity is further increased in the bipolar montages to achieve higher EF values in the lDLPFC, this will also result in stronger EF in brain areas outside the target region. To ensure control over the applied current, focalized HD-tDCS montages are recommended (Alam et al., 2016; Edwards et al., 2013), with our results showing only small variations in EF intensity when using an HD-tDCS approach. Focalizing the current meets the second criteria listed by Habich for effectively reaching the target of interest (Habich et al., 2020).

Simulation of the bipolar montages showed a more diffuse EF distribution with limited focality in both hemispheres compared to the focalized HD-tDCS montages. These results are in accordance with previous findings comparing bipolar and HD-tDCS montages in nonclinical populations (Datta et al., 2012; Laakso et al., 2016; Saturnino et al., 2015). Since AD patients seem to be more dependent on both the right and left DLPFC when executing memory tasks (Grady and Craik, 2000; Pariente et al., 2005), it is important not to inhibit the right hemisphere. In the bipolar montages, the right hemisphere is cathodally stimulated, leading to an inhibitory effect on these areas. This effect was present in all bipolar montages except the Liu montage, where the right DLPFC was stimulated anodally. In depressed patients, the montage with anodal stimulation over the lDLPFC and cathodal stimulation over the rDLPFC has been proposed to be clinically beneficial because the right hemisphere is often hyperactivated in depressed patients (Grimm et al., 2007). This is not the case for AD, where activity in the right DLPFC does not necessarily indicate disrupted processing responsible for cognitive symptoms but may instead reflect a compensatory function for preserving memory (Hill et al., 2011).

Comparison of the two HD-tDCS approaches shows that there were only minor variations in the EF distribution between the optimized and the classical F3 electrode placement. Surprisingly, the classical F3 montage had slighter stronger anodal stimulation in the target area than the optimized montage. The rule for optimizing is to choose the montage where the difference between the anodal and cathodal currents in the left DLPFC was the strongest (anodal minus the cathodal current). The analysis of anodal and cathodal hot spots in the target area shows that the classical F3 montage has a slightly higher degree of cathodal hotspots in the target area than the optimized montage. Nevertheless, the small variations present in the EF distribution are unlikely to have a strong clinical impact.

## 4 Conclusion

Several clinical trials have shown that tDCS can improve cognitive function in AD, but the results are not universally positive. A more detailed investigation of how the tDCS current interacts with cortical tissue in AD patients is necessary to enhance the chance of treatment success. Computational modeling simulates tDCS-induced current, calculating both the amount and distribution of EF in different brain regions, giving insight into how interindividual differences in brain anatomy affect tDCS stimulation.

Our results show that AD patients with disease-related neuropathology had reduced levels of EF and greater variability in current distribution than healthy matched controls. Bipolar montages with widespread EF across both hemispheres, were more affected by brain alterations in AD, compared to HD-tDCS montages where the EF was more focal to the target area. To reduce unwanted stimulation of nontarget brain areas, focal tDCS should be used. However, montage optimization for the HD-tDCS approach via individual, MR-based modeling seems to yield only modest benefits.

## Declaration of competing interest

The authors declare that they have no known competing financial interests or personal relationships that could have appeared to influence the work reported in this paper.

## Data Availability

Data will be made available on request.
