# Supplementary material for: Tracking the current in the Alzheimer's brain - Systematic differences between patients and healthy controls in the electric field induced by tDCS
Source: Neuroimage Rep. 2023 Apr 26;3(2):100172. doi: 10.1016/j.ynirp.2023.100172 (PMC12172726; doi:10.1016/j.ynirp.2023.100172)
Supplement: Multimedia component 1 [file mmc1.docx]

**Supplementary Table 1** Tissue conductivities used for modeling electric field distributions. Connectivity values from default connectivity values in SimNIBS.

| Tissue type | Conductivity (S/m) |
| --- | --- |
| Electrode sponge/gel | 1.0 |
| Skin | 0.465 |
| Skull | 0.01 |
| Cerebrospinal fluid | 1.654 |
| Gray matter | 0.275 |
| White matter | 0.126 |

**Supplementary Table 2.** Raw morphometric measurements in the Alzheimer’s and control group

| **Measure** | **Alzheimer**  **Mean (SD)** | **Healthy**  **Mean (SD)** | **F value** | **P value** |
| --- | --- | --- | --- | --- |
| *Thickness (mm)* |  |  |  |  |
| DLPFC LH | 2.26 (.12) | 2.41 (.10) | 19.68 | <.001 |
| DLPFC RH | 2.27 (.14) | 2.38 (.09) | 12.65 | .001 |
| mPFC LH | 2.48 (.14) | 2.66 (.11) | 24.00 | <.001 |
| mPFC RH | 2.49 (.19) | 2.64 (.11) | 13.75 | .001 |
| ACC LH | 2.28 (.18) | 2.41 (.14) | 7.37 | .009 |
| ACC RH | 2.25 (.16) | 2.38 (.11) | 10.81 | .002 |
| SMC LH | 2.44 (.20) | 2.63 (.12) | 15.48 | <.001 |
| SMC RH | 2.49 (.19) | 2.64 (.11) | 12.41 | <.001 |
| mOFC LH | 2.38 (.15) | 2.44 (.12) | 1.90 | .175 |
| mOFC RH | 2.38 (.21) | 2.36 (.11) | .09 | .767 |
| *Area (mm^2^)* |  |  |  |  |
| DLPFC LH | 5422,54 (656.41) | 5937.88 (860.20) | 1.488 | .024 |
| DLPFC RH | 5381.46 (1028.52) | 5861.08 (898.82) | .003 | .091 |
| mPFC LH | 3809.92 (431.19) | 4020.13 (466.29) | 2.63 | .112 |
| mPFC RH | 3942.09 (473.59) | 4262.58 (524.62) | 4.94 | .031 |
| ACC LH | 2369.88 (341.98) | 2449.13 (324.84) | .68 | .415 |
| ACC RH | 2185.29 (365.10) | 2310.92 (280.14) | 1.78 | .188 |
| SMC LH | 1631.75 (224.45) | 1665,75 (268.90) | .23 | .637 |
| SMC RH | 1454.00 (189.60) | 1463.58 (221.92) | .03 | .873 |
| mOFC LH | 1151.90 (138.66) | 1308.88 (205.78) | 9.60 | .003 |
| mOFC RH | 1475.33 (190.76) | 1534.50 (205.02) | 1.07 | .306 |
| *Volume (mm3)** |  |  |  |  |
| Total gray cortex | 390356.32 (49582.60) | 442991.82 (43252.33) | 94.715 | <.001 |
| Cortex LH | 192236.67 (23912.67) | 221014.48 (21613.27 | 98.896 | <.001 |
| Cortex RH | 198119.66 (26306.39) | 221977.34 (21712.13 | 80.695 | <.001 |
| Hippocampus LH | 2924.23 (466.79) | 3640.99 (328.91) | 27.328 | <.001 |
| Hippocampus RH | 3107.60 (576.84) | 3756.49 (378.68) | 18.450 | <.001 |
| CSF | 1371.20 (356.40) | 1036.84 (205.55) | 8.296 | .001 |

Note. LH: left hemisphere, RH: right hemisphere, DLPFC: dorsolateral prefrontal cortex, mPFC: medial prefrontal cortex, ACC: anterior cingulate cortex, SMC: supplementary motor complex, mOFC: medial orbitofrontal cortex. *Controlled for intracranial volume.

**Supplementary Table 3**. Model selection for the normfield.

| Hemisphere | Montage | Region | Group | LOOIC | $R^{2}$ |
| --- | --- | --- | --- | --- | --- |
| $\times$^a^ | $\times$^a^ | $\times$^a^ | $\times$^a^ | -30110.31 | 0.9404772 |
| $\times$ | $\times$ | $\times$ |  | -30109.49 | 0.9404890 |
| $\times$ | $\times$ | $\times$ | $\times$ | -30084.58 | 0.9416378 |
|  | $\times$ | $\times$ |  | -24126.45 | 0.8302111 |
|  | $\times$ | $\times$ | $\times$ | -24059.00 | 0.8305601 |
| $\times$ | $\times$ |  |  | -20667.72 | 0.6868933 |
| $\times$ | $\times$ |  | $\times$ | -20663.39 | 0.6876804 |
|  | $\times$ |  | $\times$ | -19605.35 | 0.6236658 |
|  | $\times$ |  |  | -19604.44 | 0.6229144 |
| $\times$ |  | $\times$ |  | -15502.55 | 0.2364756 |
| $\times$ |  | $\times$ | $\times$ | -15470.93 | 0.2386015 |
|  |  | $\times$ |  | -15190.92 | 0.1904884 |
|  |  | $\times$ | $\times$ | -15174.29 | 0.1917045 |
| $\times$ |  |  |  | -14563.37 | 0.0940515 |
| $\times$ |  |  | $\times$ | -14561.94 | 0.0944235 |
|  |  |  | $\times$ | -14422.18 | 0.0713122 |
|  |  |  |  | -14421.34 | 0.0711239 |

**Note:** ^a^ Model includes main effects only

**Supplementary Table 4.** Model selection for the CV.

| Hemisphere | Montage | Region | Group | LOOIC | $R^{2}$ |
| --- | --- | --- | --- | --- | --- |
| $\times$ | $\times$ | $\times$ | $\times$ | 34410.26 | 0.7690501 |
| $\times$^a^ | $\times$^a^ | $\times$^a^ | $\times$^a^ | 34416.96 | 0.7615191 |
| $\times$ | $\times$ | $\times$ |  | 34420.05 | 0.7614894 |
|  | $\times$ | $\times$ |  | 37751.16 | 0.5693155 |
|  | $\times$ | $\times$ | $\times$ | 37774.77 | 0.5759683 |
| $\times$ |  | $\times$ | $\times$ | 38556.29 | 0.5019029 |
| $\times$ |  | $\times$ |  | 38599.90 | 0.4949125 |
|  |  | $\times$ | $\times$ | 39791.75 | 0.3788629 |
|  |  | $\times$ |  | 39821.29 | 0.3733106 |
| $\times$ | $\times$ |  |  | 41701.02 | 0.1326741 |
| $\times$ | $\times$ |  | $\times$ | 41716.48 | 0.1351446 |
|  | $\times$ |  |  | 41921.00 | 0.0965681 |
|  | $\times$ |  | $\times$ | 41929.22 | 0.0974875 |
| $\times$ |  |  | $\times$ | 42139.97 | 0.0602239 |
| $\times$ |  |  |  | 42144.16 | 0.0588566 |
|  |  |  | $\times$ | 42289.55 | 0.0345061 |
|  |  |  |  | 42290.31 | 0.0342977 |

**Note:** ^a^ Model includes main effects, only

**Supplementary Table 5.** Model selection for the normal component (all montages).

| Hemisphere | Montage | Region | Group | LOOIC | $R^{2}$ |
| --- | --- | --- | --- | --- | --- |
| $\times$ | $\times$ | $\times$ | $\times$ | -34257.08 | 0.9408734 |
| $\times$ | $\times$ | $\times$ |  | -34250.30 | 0.9394041 |
| $\times$ |  | $\times$ |  | -23326.65 | 0.5875587 |
| $\times$ |  | $\times$ | $\times$ | -23305.65 | 0.5887291 |
|  | $\times$ | $\times$ |  | -18879.21 | 0.1270909 |
|  | $\times$ | $\times$ | $\times$ | -18760.94 | 0.1348813 |
|  |  | $\times$ |  | -18630.94 | 0.0668243 |
|  |  | $\times$ | $\times$ | -18611.93 | 0.0685313 |
|  | $\times$ |  |  | -18326.18 | 0.0142733 |
| $\times$ | $\times$ |  |  | -18316.36 | 0.0156714 |
|  | $\times$ |  | $\times$ | -18315.40 | 0.0156211 |
| $\times$ | $\times$ |  | $\times$ | -18298.23 | 0.0186140 |
|  |  |  |  | -18259.16 | 0.0001922 |
| $\times$ |  |  |  | -18257.90 | 0.0005747 |
|  |  |  | $\times$ | -18257.65 | 0.0004534 |
| $\times$ |  |  | $\times$ | -18256.99 | 0.0013824 |

**Supplementary Table 6.** Model selection for the normal component for HD-tDCS montages only.

| Hemisphere | Montage | Region | Group | LOOIC | $R^{2}$ |
| --- | --- | --- | --- | --- | --- |
| $\times$ | $\times$ | $\times$ |  | -13349.708 | 0.8787890 |
| $\times$ |  | $\times$ |  | -13339.586 | 0.8764859 |
| $\times$ |  | $\times$ | $\times$ | -13323.873 | 0.8771117 |
| $\times$ | $\times$ | $\times$ | $\times$ | -13300.059 | 0.8788613 |
|  |  | $\times$ |  | -10089.574 | 0.3267064 |
|  | $\times$ | $\times$ |  | -10075.948 | 0.3300918 |
|  |  | $\times$ | $\times$ | -10070.688 | 0.3275276 |
|  | $\times$ | $\times$ | $\times$ | -10037.725 | 0.3333147 |
|  |  |  |  | -9350.413 | 0.0005617 |
| $\times$ |  |  |  | -9348.968 | 0.0016006 |
|  | $\times$ |  |  | -9348.883 | 0.0014040 |
|  |  |  | $\times$ | -9348.664 | 0.0012783 |
| $\times$ |  |  | $\times$ | -9346.326 | 0.0030471 |
| $\times$ | $\times$ |  |  | -9345.701 | 0.0029362 |
|  | $\times$ |  | $\times$ | -9344.830 | 0.0026180 |
| $\times$ | $\times$ |  | $\times$ | -9338.884 | 0.0054867 |


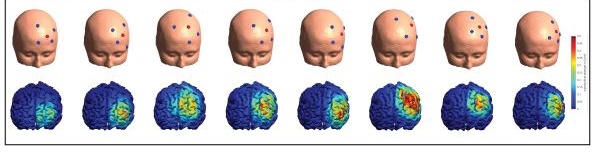
**Supplementary Figure 1.** HD-tDCS electrode montages used for individual optimization

AFF5h
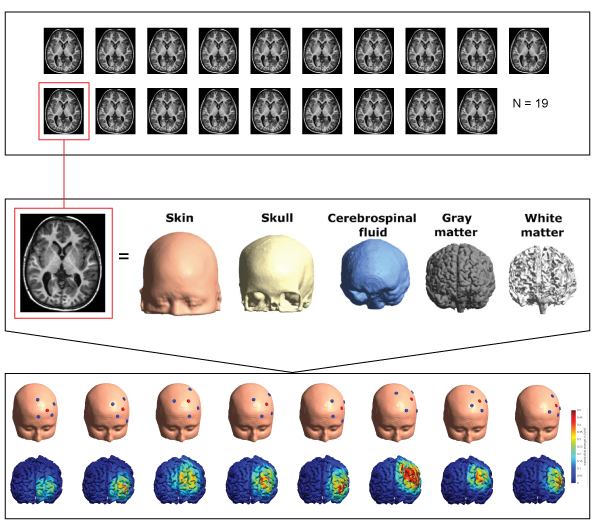


AF3
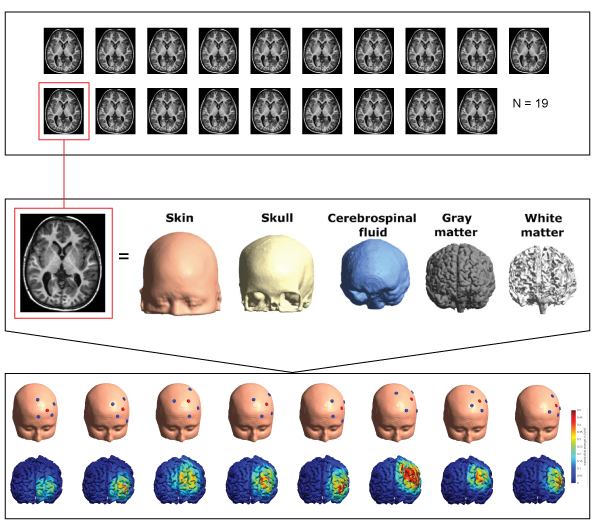


F1
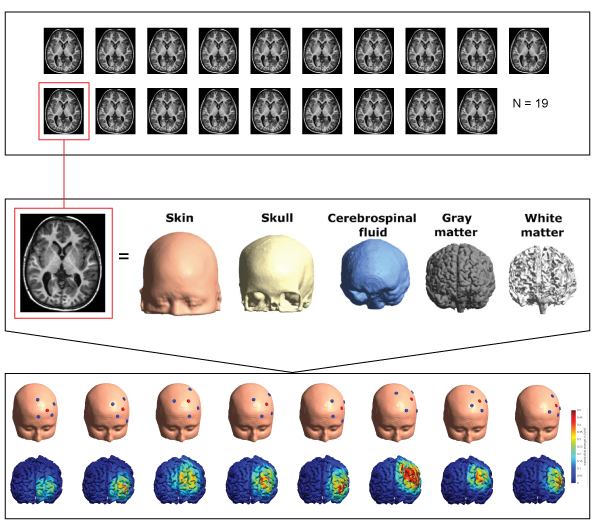


FC3
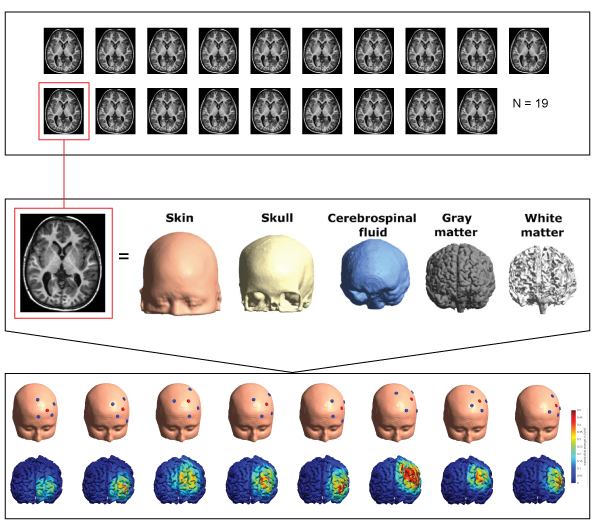


FFC5h
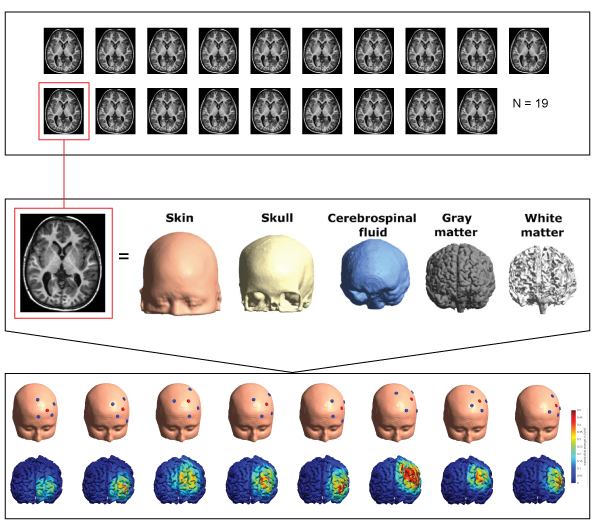


F3
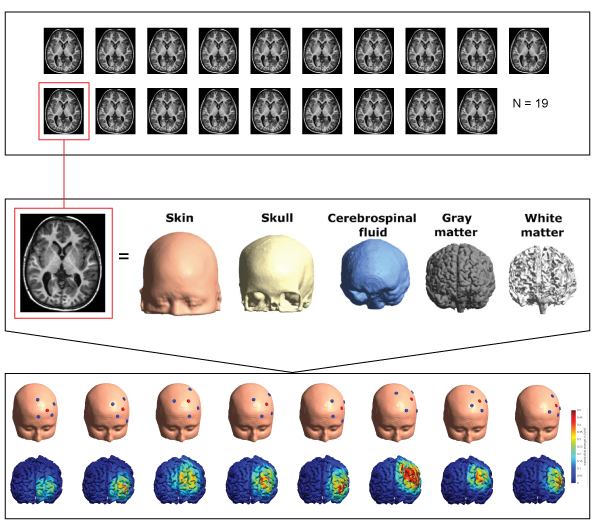


F5
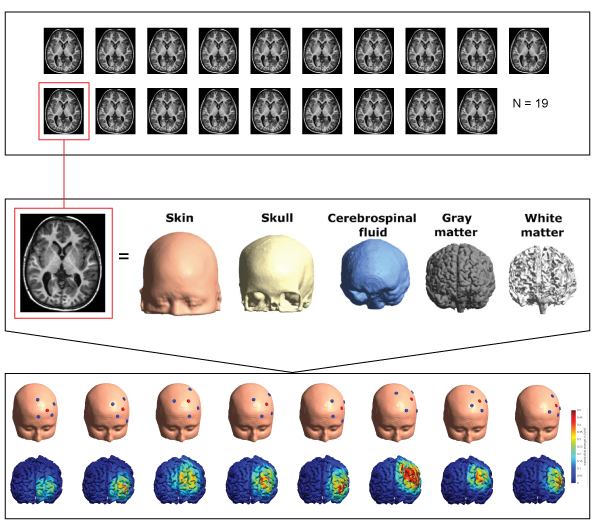


| Anode | Cathode |
| --- | --- |
| F3 | F7, C3, Fz, Fp1 |
| F5 | F9, C5, F1, Fp1 |
| FFC5 h | AF3, F7, FTT7 h, FCC3 h |
| FC3 | FT7, CP3, FCz, AF3 |
| FFC3 h | AFF5 h, FCC5 h, FCC1 h, AFF1 h |
| F1 | F5, C1, F2, Fp1 |
| AF3 | AF7, FFC5 h, Fz, Fpz |
| AFF5 h | F9, FC3, AFF1 h, Fp1 |

Note. Single, randomly selected brain model demonstrating the eight electrode placements used when choosing the optimized model for each participant.

**Supplementary Figure 2.** Coefficient of variation across all montages for the Alzheimer’s and control

**Supplementary Figure 3.** Coefficient of variation across all montages for the Alzheimer’s and control
